# Heterogeneity Assessment of Kenaf Breeding Field through Spatial Dependence Analysis on Crop Growth Status Map Derived by Unmanned Aerial Vehicle

**DOI:** 10.3390/plants12081638

**Published:** 2023-04-13

**Authors:** Gyujin Jang, Dong-Wook Kim, Won-Pyo Park, Hak-Jin Kim, Yong-Suk Chung

**Affiliations:** 1 Department of Biosystems Engineering, Seoul National University, Seoul 08826, Republic of Korea; 2 Integrated Major in Global Smart Farm, Seoul National University, Seoul 08826, Republic of Korea; 3 Department of Plant Resources and Environment, Jeju National University, Jeju 63243, Republic of Korea; 4 BrainKorea21 Global Smart Farm Educational Research Center, Seoul National University, Seoul 08826, Republic of Korea; 5 Bio-Resources and Computing Research Center, Jeju National University, Jeju 63243, Republic of Korea

**Keywords:** spatial dependence, heterogeneity, unmanned aerial vehicle, breeding, kenaf

## Abstract

The investigation of quantitative phenotypic traits resulting from the interaction between targeted genotypic traits and environmental factors is essential for breeding selection. Therefore, plot-wise controlled environmental factors must be invariable for accurate identification of phenotypes. However, the assumption of homogeneous variables within the open-field is not always accepted, and requires a spatial dependence analysis to determine whether site-specific environmental factors exist. In this study, spatial dependence within the kenaf breeding field was assessed in a geo-tagged height map derived from an unmanned aerial vehicle (UAV). Local indicators of spatial autocorrelation (LISA) were applied to the height map using Geoda software, and the LISA map was generated in order to recognize the existence of kenaf height status clusters. The spatial dependence of the breeding field used in this study appeared in a specific region. The cluster pattern was similar to the terrain elevation pattern of this field and highly correlated with drainage capacity. The cluster pattern could be utilized to design random blocks based on regions that have similar spatial dependence. We confirmed the potential of spatial dependence analysis on a crop growth status map, derived by UAV, for breeding strategy design with a tight budget.

## 1. Introduction

Kenaf (*Hibiscus cannabinus* L.) is a warm season annual fiber crop indigenous to Africa [1]. Kenaf is cultivated worldwide due to its adaptability to various climates and soil types. The usefulness of kenaf in industry and agriculture is attributed to its fibrous stems, functional compounds, and, especially, its high crude protein content [2,3,4].

The purpose of kenaf breeding is to target the development of high-yield, high-performing cultivars [5]. The height of kenaf is a representative phenotypic trait that can be selected as the breeding selection criterion to maximize the yield [6]. However, kenaf is a rapid growing plant that reaches up to 4–6 m in height, and it requires significant labor and time to measure its height in different seasons [7]. Recently, a field survey based on remote sensing has been highlighted to solve this problem with a high-throughput data acquisition system [8,9]. Unmanned aerial vehicles (UAV) provide high spatial and temporal resolution data compared to satellites and manned aircraft, enabling their use for precise spatial and temporal breeding research [10,11,12].

Phenotypic traits are determined by the interaction between targeted genotypic traits and environmental factors. Therefore, the consideration of linking the environment to genotyping is essential for breeding [13]. The assumption that there is no spatial dependence among observations except for the target treatment cannot be accepted [14,15,16]. The heterogeneity of the breeding field increases the error in the prediction of genotypic performance [17]. In order to neutralize the adverse effects from the field heterogeneity, breeders design their programs with repetition of targeted genotypes and the randomization of plot placement. However, a random design of replication for each subdivided genotype plot requires a large area and high management costs [18,19]. Moreover, the promising parental candidate is determined over several years, requiring multiple years in the same field with a recurrent phenotypic selection strategy [20]. Therefore, it is necessary to assess the heterogeneity of the experimental breeding field in order to consider the environmental error produced by spatial factors in a limited field.

Unlike general a agriculture field, in which the same treatments, such as germplasms or human factors, are applied for crop production, various treatments are applied to the breeding field for cultivar development [21]. Spatial variability of crop growth status found in the breeding field cannot directly determine the field heterogeneity because the spatial variability is a composite result of the applied treatments and environmental factors [22,23]. “Everything is related to everything else, but near things are more related that distant things” [24]. This statement is known as Tobler’s first law of geography, which Tobler invoked for emphasize of spatial analysis [25,26]. In fact, the characteristics in one position have a degree of correlation to characteristics in neighboring positions through some hidden factors. The tendency for characteristics in one area being attributed to those of the other, nearby areas is called spatial dependence [19]. Spatial dependence analysis presents objective statistics about the relationships between the growth status in a specific crop and the growth status of surrounding crops. The spatial structure in which similar crop growth is observed, regardless of the treatments applied to each plot, can be found through spatial dependence analysis. Therefore, heterogeneity in breeding field can be assessed thanks to spatial dependence analysis.

Several articles have highlighted the potential of spatial dependence analysis in the monitoring of open-field crops using crop growth maps. Ferreira et al. [27] and Paget, M.F et al. [20] selected potential parental candidates considering spatial dependence using crop growth status maps in maize and potato. They manually sampled data that were strongly correlated with crop growth status, requiring a lot of labor and time. Matese, A et al. [28] analyzed the spatial dependence between the normalized difference vegetation index (NDVI) and the volume index (VI) from a UAV crop map with agronomic variables measured in situ for precision viticulture. They found, in the context of precision agriculture, that the high NDVI area was related to low yield, and they discussed the high correlation between NDVI and sugar contents with low yield. Maimaitijiang, M et al. [29] utilized spatial dependence statistics to prove the performance of a data-driven yield prediction model. They analyzed the global degree of spatial dependence of each model, and they did not use the local degree, to find the specific site of high dependence. Despite the importance of spatial analysis and the ease of UAV based data acquisition, there has been little research on spatial dependence analysis using UAV imagery for breeding.

The overall purpose of this study is to analyze the field heterogeneity on a kenaf breeding field using a UAV-derived height map and to assess whether there are hidden environmental factors based on space in order to improve breeding strategy. The specific objectives are to (1) find heterogeneous patterns in the breeding field using spatial dependence analysis, (2) reveal the reason for the existence of spatial dependence in this experimental breeding field, and (3) discuss a breeding strategy in a limited field with a tight budget.

## 2. Results

### 2.1. The Reliability of Crop Height Map

The $R^{2}$ and RC of the linear equation between the estimated and the actual height were 0.80 and 0.94, respectively. The result shows a strong correlation between the estimated height and the actual height, and it was acquired according to high $R^{2}$ and RC. Due to the strong linear relationship, the height map of the various growth stages can be used for the assessment of spatial dependence. In the analysis of crop height, the performance of $R^{2}$ is estimated as high ($R^{2}$ > 0.7), medium (0.7 > $R^{2}$ > 0.5), and low ($R^{2}$ < 0.5), and the acquired $R^{2}$ in this study is ‘high’ [30]. This result is similar to previous studies that estimated the height using this method. This method was applied to various crops, such as rice [31], barley [30], maize [32], and wheat [33] with kenaf-like shape (long and thin) and performed reasonably. The UAV-derived height map can be utilized for crop growth status detection with acceptable reliability for crop height estimation, and it shows a high correlation between height and yield in kenaf [6].

### 2.2. The Recognition of Cluster Region in Local Indicators of Spatial Autocorrelation (LISA) Map

**Figure 1:**
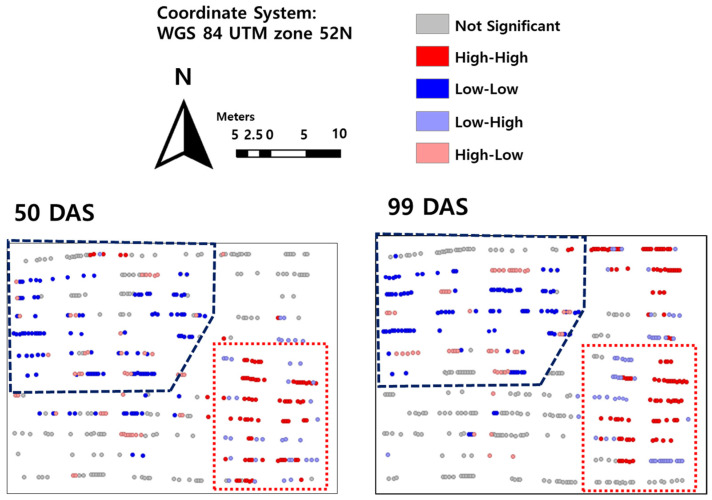
The Local indicators of Spatial autocorrelation cluster map (LISA) in 50 and 99 days after sowing. Blue dot and red dot are representative L-L and H-H, respectively. Moreover, blue dot polygon and the red dot polygon are indicated low-growth region and high-growth region, respectively.

In two growth stages, the cluster regions, expressed as dot polygons, appeared to be similar. The low-growth region (blue dot polygon) and the high-growth region (red dot polygon) appeared in the northwest and in the southeast, respectively (Figure 1). A similar pattern from the early to later growth stages might imply that environmental factors based on space were applied consistently.

The similarity analysis among the germplasms in the cluster region is displayed in Table 1 and Table 2. Six plots in the low-growth region and two plots in the high-growth region, and ten plots in low-growth region and six plots in high-growth region, were not significantly different at 50 DAS and at 99 DAS. Although there are some germplasms that did not group with the cluster region, most of germplasms were coincident with the cluster region. In particular, the germplasms placed in both the high- and low-growth regions resulted in a high difference (Table 1 and Table 2). Finally, we can conclude, by recognition of cluster region by LISA map, that spatial dependence existed in the breeding field utilized in this study. 

### 2.3. The Reason of Field Heterogeneity: Slope of High-Clay Field

**Figure 2:**
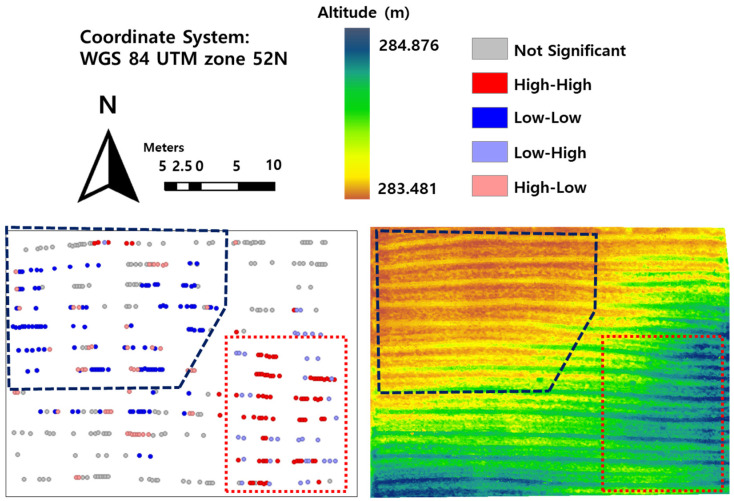
The comparison of cluster patterns in the LISA map and terrain elevation patterns of the altitude map in the breeding field. The blue dotted polygon in the LISA map and the terrain elevation map is representative of the low-growth region and the low-altitude region, respectively. The red dotted polygon in the LISA map and the terrain elevation map is representative of the high-growth region and the high-altitude region, respectively.

In order to minimize the heterogeneity of the breeding field in this study, all of the human factors, such as tillage impact, fertilizer and pesticide inputs, and irrigation amount, were applied uniformly across the whole field. Therefore, it is difficult to conclude that field heterogeneity was the result of human crop management. In this context, we shifted our focus to elevation, which showed a pattern similar to that of the LISA map, in order to find the reason (Figure 2).

The texture of the breeding field is silty clay loam with approximately 30% clay content (Table 3). High content of clay and compaction of the subsoil reduce soil percolation, delaying water drainage time [34,35,36]. It should be noted that water drainage delay can negatively affect crop growth.

The soil moistures were 24.78 ± 1.16% and 22.13 ± 2.91% in the low-altitude region and the high-altitude region, respectively. As result of the statistical test, the null hypothesis, in which the soil moisture in the low-altitude region was higher than that in the high-altitude region, was not rejected (*p*-value > 0.05). Thus, the soil moisture in low-altitude regions was significantly lower than that in high-altitude regions. From this result, it is reasonable to assume that water from rainfall or irrigation flows down to the lower altitude area following the slope.

This breeding field has a drainage issue due to the high-clay soil. Since the field has a slope in a downward direction to the northwest (Figure 2), excessive moisture conditions may be formed in the northwest region due to poor drainage. Moreover, the low growth of kenaf was observed to be related to overwatering in previous research [37]. To integrate these facts, the growth status of kenaf, which is sensitive to high moisture conditions, was observed to be low in the overwatered northwest region due to precipitation and a northwest slope of high-clay field. Therefore, we can conclude that the reason for field heterogeneity is attributed to the elevation pattern in the high-clay field.

### 2.4. Breeding Strategy Considering Heterogeneous of Breeding Field

**Figure 3:**
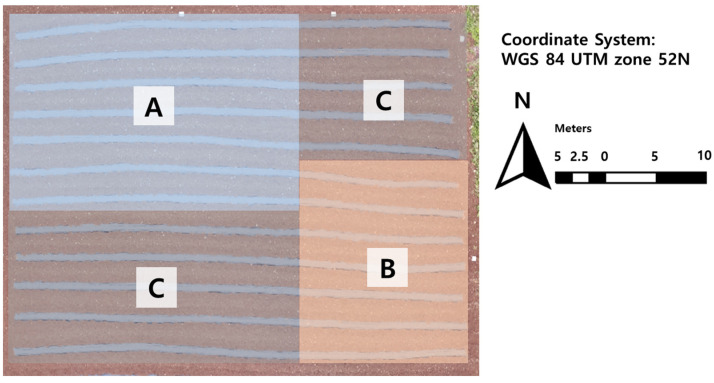
Heterogenous blocks in the breeding field in this study.

Through the spatial dependence analysis on the breeding field, the field heterogeneous blocks can be divided as in Figure 3. Regions A and B represent regions with low and high crop growing conditions, respectively. Region C is invariable within the whole field, compared to other regions, with high randomness from the LISA map. Neutralizing the field heterogeneity for breeding can be achieved by designing separate blocks between the A, B, and C regions. Since each region provides spatially similar environmental factors, the repetition of germplasms can be decreased, and the variety of germplasms can be increased in a tight field by separate breeding selection from each region.

## 3. Discussions

The main goal of this research was to assess the heterogeneity in a breeding field, using a UAV-derived crop growth status map, to improve breeding block design. Local indicators of spatial autocorrelation (LISA) were utilized for spatial dependence analysis. Low-growth regions and high-growth regions were recognized in the northwest and in the southeast areas of the breeding field, respectively. Based on the similarity between the elevation pattern and the LISA map pattern, the result of field surveys, soil moisture, and soil texture measurements revealed that the field heterogeneity was due to a northwest slope direction combined with a high-clay field.

Because terrain factors, soil texture, and elevation are not changeable in this study, field heterogeneity in moisture must be maintained continuously. Soil moisture is an important variable in crop growth [38,39,40,41]. Therefore, uniform moisture conditions are required across the open-field for breeding. Precision irrigation is a kind of precision agriculture technique, and it performs irrigation supply in a site-specific method based on the spatially variable water requirement within the field [42,43,44]. However, the costs of precision irrigation systems are high because they require sensors, such as soil moisture sensors or thermal imaging sensors, data monitoring servers, and irrigation equipment capable of spatially precise control [45,46].

Spatial dependence analysis is a field of study that seeks to understand how the spatial relationships between data points affect their behavior [25,47]. This field has many applications in various disciplines, including geography, ecology, economics, and epidemiology, among others [48,49,50,51,52]. The prospects for spatial dependence analysis are quite promising, given the increasing availability of spatial data and the advances in computing power and statistical methods [53,54]. They include the following: First, it helps improve data collection and processing [48,55]. With the increasing availability of remote sensing and geolocation technologies, it is becoming easier to collect and process large amounts of spatial data. This is likely to lead to more comprehensive and accurate analyses of spatial dependence. Second, it integrates with machine learning [56,57]. Machine learning algorithms can be used to identify complex patterns in spatial data that may be difficult to discern using traditional statistical methods. Combining machine learning with spatial dependence analysis could lead to more accurate predictions and insights. Third, it can be applied to interdisciplinary applications [58,59,60]. Spatial dependence analysis has many potential applications across various fields, including public health, transportation planning, and environmental management. As researchers in these fields continue to recognize the importance of spatial relationships, the demand for spatial dependence analysis is likely to grow. Fourth, it is useful for the development of new techniques [61,62]. Spatial dependence analysis is a relatively young field, and there is still much to learn about how spatial relationships affect data behavior. As researchers continue to develop new techniques for analyzing spatial data, the field is likely to grow and evolve.

In this research, we suggested a uniform management method of breeding to reduce the environmental error discussed in Section 2.4. Management zones that have spatially similar environmental factors can be constructed based on spatial heterogeneous patterns acquired from UAV-derived crop growth maps. The random block design of experimental germplasms is separately applied in each management zone. Replication can be reduced in this method. For instance, through this study, 24 germplasms can be placed in any management zone (A, B, or C in Figure 3). We can utilize only one zone for 24 germplasms and add more varieties of kenaf germplasm. Moreover, the UAV mounted RGB sensor was the only additional equipment, compared to the original breeding experiment, and the whole-field data could be acquired with only one flight in each growth stage. Spatial dependence analysis using a UAV data acquisition system can be suggested as a cost-effective and labor-effective method for uniform field management for breeding. However, this method can be only be applied after the crop grows. Thus, we recommend pre-measurement of terrain factors such as soil texture, soil electrical conductivity, and elevation. These terrain factors are important influences on field heterogeneity, as evidenced by this research. The acquired data may be useful in the construction of management zones.

In the future, we will compare environmental error terms in the data from 2019, and we will apply this method to validate a suggested breeding strategy. Moreover, this method will be another open-field analysis and will be generally available.

## 4. Materials and Methods

### 4.1. Study Site and Field Characteristics

The study was conducted during the 2019 growing season (May to August) in a breeding field located in Ara1-dong, Jeju-si, Jeju-do, Republic of Korea (33°27′33″ N 126°33′23″ E, altitude: 284 m). During the experiment, the average temperature ranged from 14.7 °C to 31.1 °C, and the total amount and the frequency of precipitation were recorded as 967.1 mm and 50, respectively. Moreover, the breeding field was subjected to typhoons with high precipitation (DANAS, LEDIMA, KROSA) three times during the experiment.

**Figure 4:**
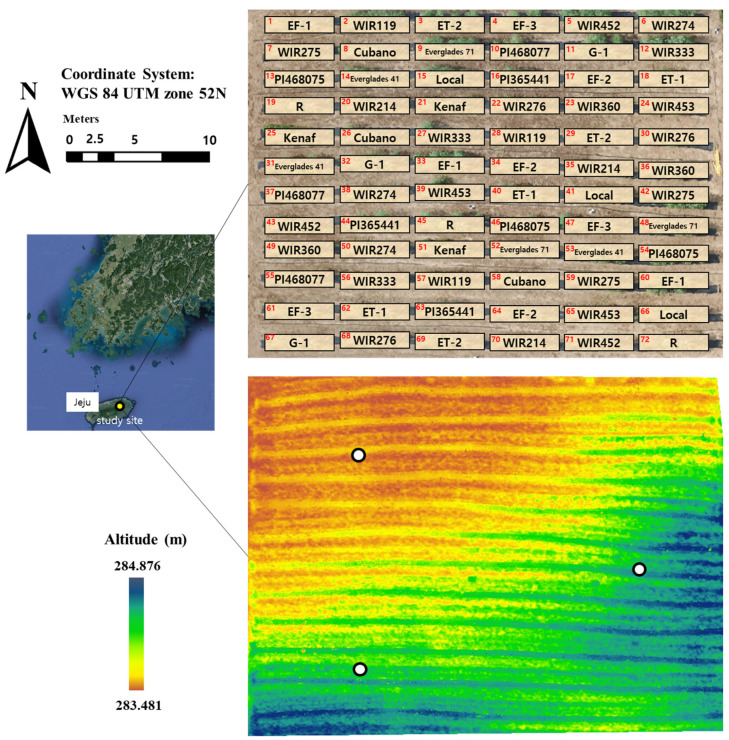
Breeding field used in this study. The upper map shows breeding design, and the letters and the numbers marked in red in each box indicate the varieties of germplasms and plot labels, respectively. The lower map shows the terrain elevation of this field. Three white circles within the field are the soil sampling locations.

Three soil samples from randomly distributed points within the field were collected with an auger at two depths (0–20 and 20–40 cm) (Figure 4). The collected soil samples were air-dried and passed through a 2 mm sieve for analysis. The selected soil properties were analyzed according to the analysis methods for soil physical properties [63]. After removing organic matter, the sand, silt, and clay contents were measured by wet sieving (sand fractions) and pipette (silt and clay) methods. Bulk density was calculated using 100 cm^3^ of undisturbed core samples dried at 105 °C.

The pattern of the terrain altitude of the breeding field was acquired using UAV imagery and the kenaf height calculation process (Figure 4). In the next section, details for the terrain altitude generation procedure are given. The altitude is high in the southeast and low in the northwest with a slope in the northwest direction (Figure 4). The slope of the field can produce a difference in soil moisture content between low-altitude and high-altitude areas. Therefore, we collected four soil samples, from 10 to 20 cm deep, from each region after rain using a soil sampling ring kit (Eijkelkamp, Gelderlan, The Netherlands). The gravimetric water content (%) of the soil samples was determined by the oven drying method. A non-parametric test (Wilcoxon rank sum test) was applied to compare the soil moisture of each region, considering no assumptions about the normal distributions due to a low sampling number. Statistical analysis was performed using R software (R. 4.1.2, RStudio Team, R Foundation for Statistical Computing, Boston, MA, USA). Soil moisture was measured by a standard soil–water testing laboratory (National Instrumentation for Environment Management (NICEM), Seoul, Republic of Korea).

### 4.2. Plant Preparation

Seeds were provided by the National Gene Bank of the Rural Development Administration (RDA), Republic of Korea (http://genebank.rda.go.kr/eng/uat/uia/actionMain.do, accessed on 1 January 2022). The study site design constituted 3 plot replications of 24 germplasms for a total of 72 plots (Figure 4). On 5 May 2019, all cultivars were direct-sowed at 15 points 25 cm apart within a plot, and, in total, 72 plots of each cultivar were randomly distributed across the whole field at a distance of 50 cm. Ridges and furrows were built in the field, and all of the seeds were planted on a ridge to avoid damage from the drainage problem. After 15 days of sowing, irrigation, using a perforated hose, was performed once per day until the harvest. For the reduction of adverse effects from weeds, black plastic mulch films were covered on each row. Additionally, pesticide and fertilizer were consistently applied across the whole field. Because the ‘EF-2’ did not germinate, while the germination of the other cultivars was generally 70~80%, the plots were regarded as bare ground.

### 4.3. The Generation of Height Map Using UAV-RGB

A MAVIC Zoom 2 (SZ DJI Technology Company, Shenzhen, China), with mounted global shutter RGB sensors capable of producing images with a resolution of 12-megapixels (4000 × 3000), captured multiple overlapped images during automatic fly tracking based on pre-set waypoints at an altitude of 40 m using Pix4Dcapture (Pix4D SA, Lausanne, Switzerland). The UAV fights were carried out three times for the investigation of the crop growth status. One image capture was conducted immediately after sowing, when the crop was not germinated, for the generation of the digital terrain model (DTM) of the breeding field (Table 4).

Although a georeferencing of map can be created using GPS tagged to images synchronized to the images from the UAV, the accuracy of the geospatial data on the generated map was not enough for spatial analysis due to the instability of flying [64,65]. For more accurate geo-referencing, ten ground control points (GCPs), made in easily recognized checkboard form and including accurate geospatial data based on a real-time kinematic-global system (RTK-GPS), were placed across the whole field and were utilized for geometric calibration of the image mosaicking process. The GCPs coordinates were measured with a C099-F9P receiver (u-blox, Thalwil, Switzerland) with a horizontal and vertical precision of 0.01 m.

All of the captured RGB images were processed in the software Pix4Dmapper (Pix4D SA, Lausanne, Switzerland) based on the structure from motion (SfM) algorithm, and we generated an orthoimage and digital surface model (DSM) with a resolution of 1.4 cm per pixel. In order to generate the height map, DSM, which is represented by the crop surface, was subtracted from DTM, as in a previous study [30,66,67].

### 4.4. The Extraction of Individual Height

In this study, spatial dependence for crop growth status was investigated through the analysis of height distribution in the field. The crops were evenly distributed across the field in all flight campaigns, except for ‘EF-2’ plots, which were an area of no germination. Thus, individual height extraction was required.

**Figure 5:**
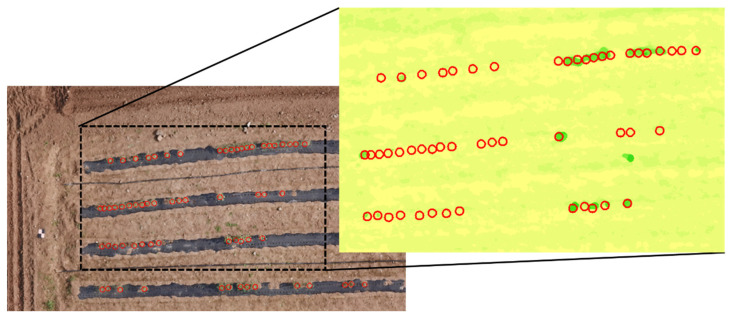
An example of individual height extraction. Based on the crop position from the RGB orthoimage in initial growth stages, the circles were generated. The circles were utilized in all of the growth stages.

It was difficult to extract the individual height at 99 days after sowing (DAS) because the short distance between the sowing points caused overlap among the adjacent individual. Fortunately, in the initial growth stages (50 DAS), only a little overlap occurred among the adjacent individuals, and the individual center positions were delineated within the orthoimages manually. From each center point, a 40 cm diameter circle was drawn using the ‘Create Buffer Zone’ function in ENVI software, and we extracted the max value from the height map of every growth stage (Figure 5). Extracted values were normalized, and we removed values lower than −1.646, i.e., below 5% following the cumulative probability, as outliers [31,68].

For evaluation of the generated height map, the correlation between the estimated kenaf height, according to the height map, and the actual kenaf height, measured using a ruler, were calculated. The regression coefficient (RC) and the coefficient of determination ($R^{2}$) were utilized as indicators of high correlation. The maximum value extracted from each plot in the height map represented the estimated kenaf height, and the height of the highest kenaf within the plot represented the actual kenaf height. The estimated height was extracted using ENVI (L3 HARRIS Geospatial, Broomfield, CO, USA) software. After validation of the plant height estimation method, the plant height value of each individual was regarded as the crop growth status in this study.

### 4.5. Spatial Dependence Analysis of Breeding Field

As mentioned in the above section, three plot replications for twenty-four cultivars were distributed randomly across the whole field. It is reasonable to conclude that the height of the crop is significantly different from that of crops grown outside the plot due to the differences in germplasms. However, if the individual height of the crops from different plots are similarly high or low, spatial dependence can be suspected to exist within the field, except for the case in which all of the cultivars in the spatial-dependence-showing region are the same. In this study, spatial dependence was assessed through analysis of whether there were clusters with high or low crop heights beyond the plot area.

Spatial autocorrelation statistics enable to measure the degree of spatial dependence with numerical objectivity [25]. In this study, local indicators of spatial autocorrelation (LISA) were used as a spatial autocorrelation statistics tool [69]. LISA analysis can provide an overall patterns of the target area, including the existence of clusters and their locations. LISA analysis was carried out on a scatterplot between the normalization of the observed variable and the spatial lag calculated as average value of the weighted variable in a nearby location, as shown in Equation (1).

(1) $$I_{i}=\frac{Z_{i}}{m_{i}}\;\underset{j}{\sum }W_{ij}Z_{j}\;,\mathrm{where}\;m_{i}=\frac{\sum Z_{i}^{2}}{N}$$

The equation is the local Moran statistics calculation, where $Z_{i}$ is the normalization of the variable and $W_{ij}$ is the spatial weight matrix between location *i* and *j*. The positional relationship among the points (neighbors) are determined by various positional setting algorithms, such as the inverse distance method or the contiguity method. The weight matrix of each point was represented by 1 for a neighbor and 0 for a non-neighbor.

**Figure 6:**
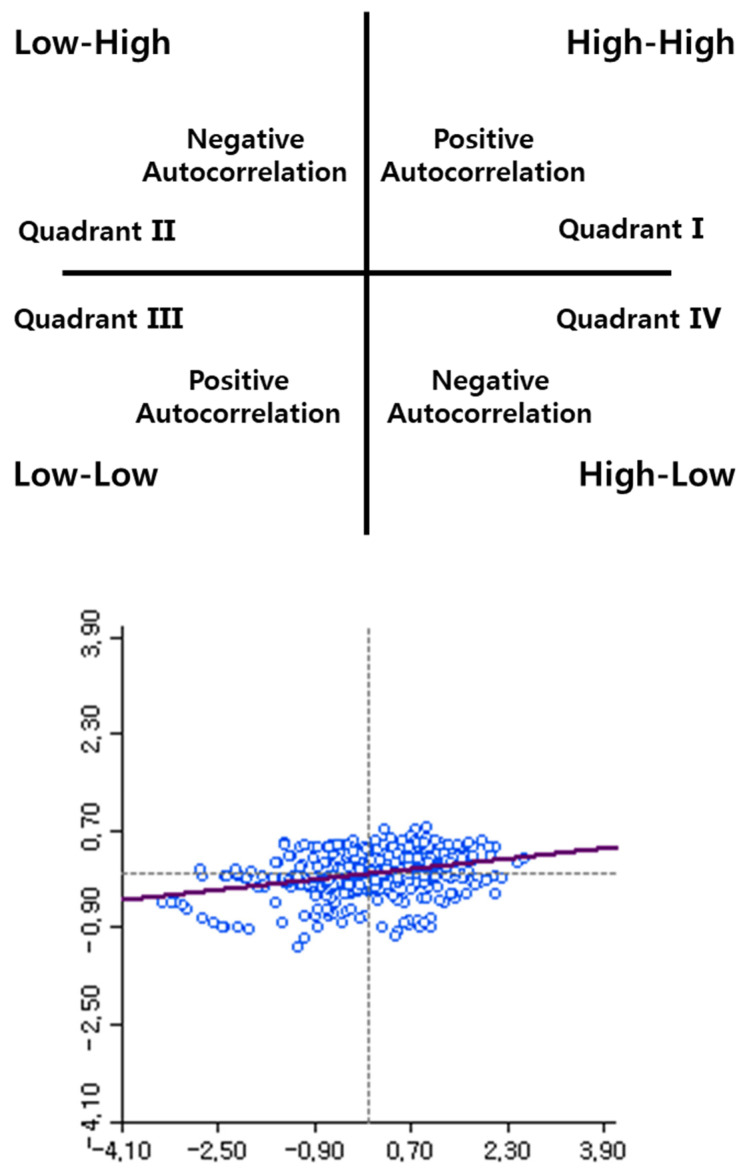
The scatterplot showing the relationship between the variables corresponding to the observed point and spatial lag (**upper**). An example of a scatterplot (**lower**). The *x*-axis and *y*-axis are representative of the observed crop growth status of one individual, and spatial lag calculated by Equation (1). This scatterplot was made from all observed individual crop growth statuses.

The four quadrants on the scatterplot showing the relationship between the variable and the spatial lag, as well as an example scatterplot, are illustrated in Figure 6. Four quadrants are indicated in the spatial class. (a) Quadrant 1: individual with high crop growth status and neighboring individuals with high crop growth status (H-H), (b) Quadrant 3: individual with low crop growth status and neighboring individuals with low crop growth status (L-L), (c) Quadrant 2: individual with low crop growth status and neighboring individuals with high crop growth status (L-H), and (d) Quadrant 2: individual with high crop growth status and neighboring individuals with low crop growth status (H-L). In this study, the regions of H-H and L-L were highlighted for finding the spatial dependence of the breeding environment. The regions with intensively distributed H-H and L-L clusters were denoted as the high-crop-growth region and the low-crop-growth region, respectively, and the integral was expressed as the cluster region.

The spatial class of each observed variable position was determined by a test of significance and the Monte Carlo random permutations among the observed values. The pseudo *p*-value that resulted from each test assessed the significance of whether there was spatial dependence on a specific position. In conclusion, the spatial pattern (H-H, L-L, L-H, H-L, and randomness) of each position was attributed to a pseudo *p*-value and the location within the scatterplot (Figure 6).

A process of LISA analysis was conducted using GeoDa software (Ver 1.20, Chicago University, Chicago, IL, USA). The confidence level for rejection of the null hypothesis of spatial randomness was set to 99%, with a pseudo *p*-value of 0.01, through a computation approach based on 999 random permutations. All individuals within the plot were regarded as having at least one neighbor from the other plot. Thus, the spatial weight matrix was set based on the inverse distance method using a distance of 1.75 m, considering that the farthest distance between adjacent plots is 1.75 m.

Moreover, considering the random distribution of three replications for each germplasm, the pattern of the LISA map can be attributed to the original crop growth capability of germplasm, not environmental factors based on space. Therefore, individual heights of the germplasm in the cluster region were compared with those in other regions. Non-parametric tests (Kruskal–Wallis test, post hoc Dunn’s test with Benjamini–Hochberg FER correction) were applied to compare the heights of the three plots. Statistical analysis of the difference between regions can explain whether the spatial dependence is due to spatial factors. Data analysis was performed using R software (R, 4.1.2, RStudio Team, R Foundation for Statistical Computing, Boston, MA, USA).

## Figures and Tables

**Table 1:** The results of Kruskal–Wallis test among the replications. All plots in this table are in the high-growth region (red dot polygon in Figure 1).

Plot Number	Germplasm	*p*-Value at 50 DAS ^1^	*p*-Value at 99 DAS
1	EF-1	*	*
2	WIR119	*	0.175
3	ET-2	*	0.273
4	EF-3	0.375	0.635
7	WIR275	**	**
8	Cubano	0.372	0.706
9	Everglades 71	**	*
10	PI468077	*	*
13	PI468075	**	**
14	Everglades 41	**	*
15	Local	*	0.103
16	PI365441	*	**
19	R	*	0.157

^1^ Days after sowing. * and ** indicate significance at *p*-values of 0.1 and 0.01.

**Table 2:** The results of Kruskal–Wallis test among the replications. All plots in this table are in the low-growth region (blue dot polygon in Figure 1).

Plot Number	Germplasm	*p*-Value at 50 DAS ^1^	*p*-Value at 99 DAS
1	EF-1	*	*
2	WIR119	*	0.175
3	ET-2	*	0.273
4	EF-3	0.375	0.635
7	WIR275	**	**
8	Cubano	0.372	0.706
9	Everglades 71	**	*
10	PI468077	*	*
13	PI468075	**	**
14	Everglades 41	**	*
15	Local	*	0.103
16	PI365441	*	**
19	R	*	0.157
20	WIR214	0.157	*
21	Kenaf	*	**
22	WIR276	*	*
25	Kenaf	*	**
26	Cubano	0.372	0.706
27	WIR333	0.105	*
28	WIR119	*	0.175
31	Everglades 41	**	*
32	G-1	0.106	0.753
33	EF-1	*	*
37	PI468077	*	*
38	WIR274	*	*
39	WIR453	*	0.204

^1^ Days after sowing. * and ** indicate significance at *p*-values of 0.1 and 0.01.

**Table 3:** The soil properties of the breeding field in this study.

Parameters	Topsoil (0–20 cm)	Subsoil (20–40 cm)
Bulk density (g/m^−3^)	1.23 ± 0.07	1.47 ± 0.17
Soil texture	Silty clay loam	Silty clay loam
Clay (%)	30.6 ± 0.23	29.2 ± 1.15
Silt (%)	56.2 ± 2.15	58.9 ± 0.60
Sand (%)	13.2 ± 1.95	11.9 ± 1.60

**Table 4:** The campaign of the unmanned aerial vehicle used in this study.

Date	Flight Altitude (m)	Ground Sampling Distance (cm/pixel)	Wind (m/s)	Image Overlap (%)
26 April 2019	30	0.65	4.4	>80
22 June 2019 (50 DAS ^1^)	40	1.41	3.9	>90
6 August 2019 (99 DAS)	40	1.34	3.2	>90

^1^ Days after sowing.

## Data Availability

Not applicable.
